# Comparison of Ethanolic and Aqueous-Polyethylenglycolic Propolis Extracts: Chemical Composition and Antioxidant Properties

**DOI:** 10.1155/2021/5557667

**Published:** 2021-03-17

**Authors:** Mindaugas Liaudanskas, Loreta Kubilienė, Vaidotas Žvikas, Sonata Trumbeckaitė

**Affiliations:** ^1^Department of Pharmacognosy, Faculty of Pharmacy, Medical Academy, Lithuanian University of Health Sciences, Sukilėlių Av. 13, LT-50162 Kaunas, Lithuania; ^2^Institute of Pharmaceutical Technologies, Faculty of Pharmacy, Medical Academy, Lithuanian University of Health Sciences, Sukilėlių Av. 13, LT-50162 Kaunas, Lithuania; ^3^Department of Drug Technology and Social Pharmacy, Faculty of Pharmacy, Medical Academy, Lithuanian University of Health Sciences, Sukilėlių Av. 13, LT-50162 Kaunas, Lithuania; ^4^Neuroscience Institute, Medical Academy, Lithuanian University of Health Sciences, Sukilėlių Av. 13, LT-50162 Kaunas, Lithuania

## Abstract

In recent years, particular attention has been paid to the natural antioxidants. Bee products, especially propolis, are characterized by multifunctional (antioxidant, anti-inflammatory, antibacterial, antiviral, and food preservative) effects and can be used for the development of functional food or food preservatives. Propolis extracts that are commonly produced are ethanolic; therefore, to certain groups of consumers, for example, children and alcohol sensitive group, their applicability is limited. The aim of this study was to develop alternative propolis from aqueous-polyethylenglycolic propolis extract (AQUA-PEG) and compare the chemical composition as well as antioxidant (radical-scavenging and reduction properties) activities to those of ethanolic propolis extract (EEP). Polyethylene glycol is quite a good solvent, which can be successfully used for the preparation of NEP. The total quantity of phenolic compounds identified in AQUA-PEG (400.36 *µ*g/mL), prepared according to our technology, is very similar to that of EEP (433.53 *µ*g/mL), whereas the amount of phenolic acids was greater by 1.31-fold in AQUA-PEG and of flavonoids was greater by 2.38-fold in EEP. The antioxidant activity depends on the method used: by applying the ABTS and CUPRAC methods, both extracts demonstrate similar antioxidant (antiradical and reducing) activity, whereas in the case of the DPPH and FRAP method, significantly higher antioxidant activity was detected in EEP. This should be taken by researchers into account especially when interpreting the results and drawing conclusions about the antioxidant activity of propolis extracts. On the basis of the results, AQUA-PEG, prepared by the developed technology, can be used as an alternative form to ethanolic propolis extract, since it contains a large quantity of antioxidants, namely, flavonoids and phenolic acids. We believe that nonethanolic propolis extract has the prospect of being applied for the development of functional foods in order to alleviate certain symptoms of oxidative stress or for the prevention of some oxidative-stress-related diseases.

## 1. Introduction

Consumers, who care about their health, choose food products that not only provide the body with the necessary nutrients but also help protect against illnesses. Food may be enriched with various components, such as vitamins, minerals, antioxidants, and amino acids. Such food products differ from conventional ones in their biological value and are meant to satisfy specific bodily functions. Such functional food has a positive effect on one or more bodily functions due to constituents that improve health. Bee products, especially propolis, are characterized by multifunctional (antioxidant, anti-inflammatory, antibacterial, and antiviral) effects and can become a useful raw material for the development and enrichment of such products. Moreover, propolis has certain properties as a food preservative which are ensured by the bactericidal and bacteriostatic properties of propolis [1]. This is especially relevant in the food industry when it comes to searching for natural preservatives. In recent years, particular attention has been paid to the search for natural antioxidants. It is important to note that the main constituents of propolis show relatively strong antioxidant activity, which also correlates with the total content of polyphenols and flavonoids [2]. Furthermore, most components of propolis are natural constituents of food products and are recognized as safe substances. The problem remains the solubility of propolis active substances as they are mainly soluble in ethanol.

Therefore, propolis extracts that are commonly produced and used are ethanolic, which means that wider application thereof to certain groups of consumers, for example, children with chronic diseases and alcohol sensitive group, due to possible drug interactions, is limited. Consequently, more and more studies on the development of nonethanolic propolis extracts are being conducted in practice in order to develop an equally or more effective propolis extract as ethanolic propolis extract.

It has been already revealed that nonethanolic propolis extract possesses antibacterial [3], antiviral [4], and anti‐inflammatory [5] properties. It has also been reported that nonethanolic propolis extract protects against cell damage induced by ionizing radiation [6]. The new findings provide evidence that the protective effect of nonethanolic propolis extracts against intestinal radiation damage involves not only its anti-inflammatory and antioxidant effects but also its antiapoptotic properties as well [7]. Studies confirm that nonethanolic propolis extract is potentially effective as an adjuvant to therapy in asthmatic patients. The benefits may be related to the presence of caffeic acid derivatives and other active constituents in the extract [8].

One fundamental aspect of the development of alternative propolis forms is its solubility, since approximately 50–70% of propolis mass may be dissolved in 70% ethanol and only up to 5% in water [9]. Therefore, the selection of solvent is one of the most important stages of the development of a successful technology for the preparation of nonethanolic propolis extract. As polyethylene glycol (PEG) is widely used as a solvent and preservative in many nonparenteral and parenteral forms of medicines for the improvement of poor water solubility, ethanolic propolis extract (EEP) and aqueous-polyethylenglycolic propolis extract (AQUA-PEG) were prepared for the purposes of this study. The question was whether AQUA-PEG is as effective as EEP in terms of their biological effect and chemical composition. Thus, the aim of this study was to determine and compare the chemical composition and antioxidant (radical-scavenging and reduction properties) activity of EEP and AQUA-PEG. Four different methods (DPPH, ABTS, FRAP, and CUPRAC) for determining the antioxidant activity of EEP and AQUA-PEG as well as comparing the advantages and disadvantages of the methods were used.

## 2. Materials and Methods

### 2.1. Reagents

Distilled water was purified using the Milli-Q system (Millipore, Bedford, MA, USA). Ethanol (96%) was obtained from Vilniaus Degtine (Vilnius, Lithuania). Acetonitrile (LC-MS grade), formic acid for LC-MS, 2,2′-azino-bis (3-ethylbenzothiazoline-6-sulfonic acid (ABTS), potassium persulfate (K_2_S_2_O_8_), 2,2-diphenyl-1-picrylhydrazyl (DPPH), sodium acetate trihydrate, anhydrous acetic acid (99.8%), hydrochloric acid (37%), iron (III) chloride hexahydrate (FeCl_3_ × 6H_2_O), 2,4,6-tripyridyl-s-triazine (TPTZ), copper (II) chloride dihydrate (CuCl_2_ × 2H_2_O), ammonium acetate, and neocuproine were acquired from Sigma-Aldrich (St. Louis, MO, USA). All standards used for UPLC-ESI-MS/MS analysis were also purchased from Sigma-Aldrich (St. Louis, MO, USA), except for isorhamnetin 3-O-glucoside, which was obtained from Extrasynthese (Genay, France).

### 2.2. Propolis Raw Material and Preparation of Propolis Extracts

Raw propolis was obtained from JSC Bitute (Vilnius, Lithuania). Prior to the analysis, the propolis samples were kept at room temperature in the dark. Preparation of EEP: the EEP was made by adding ethanol (70%) (1 : 10 (w/v) sample to solvent ratio) to propolis ground into powder. The extraction was performed at room temperature for 72 hours by shaking. Then the EEP was filtered through Whatman No. 1 filter paper and left at the refrigerator (7°C) for 24 hours to separate the wax. After that, the EEP was filtered again through Whatman No.1 filter paper and used for experiments. Preparation of AQUA-PEG: raw propolis was ground into powder. The AQUA-PEG was prepared by adding a system of solvents, namely, a mixture of water and 30% polyethylene glycol 400 (macrogol), at 1 : 10 (w/w) sample to solvent ratio. Extractions were conducted for 10 min at 70°C temperature in an ultrasonic bath of 35 kHz frequency. Both solutions are clear yellow liquids, which remain stable at the time of storage; that is, the color remains unchanged; no precipitate is observed; and they do not turn white. Prior to the UPLC-ESI-MS/MS analysis, the extract was filtered through membrane filters (pore size 0.22 *µ*m) produced by Carl Roth GmbH (Carl Roth GmbH & Co. KG, Karlsruhe, Germany).

### 2.3. UHPLC-ESI-MS/MS Conditions

The qualitative and quantitative analysis of phenolic compounds was performed according to the previously validated and described UHPLC-ESI-MS/MS method [10]. The mass spectrometry parameters for the analysis of phenolic compounds and quinic acid are presented in Table 1.

### 2.4. Evaluation of Antioxidant Activity

#### 2.4.1. DPPH Method

The DPPH solution in 96.3% v/v ethanol (3 mL, 6 × 10^−5^ M) was mixed with 10 *μ*L of the extracts. A decrease in absorbance was determined at *λ* = 515 nm using a double beam UV/VIS spectrophotometer M550 (Spectronic CamSpec, Garforth, UK) [11]. This spectrophotometer was employed in the case of all antioxidant-related experiments. The results of all antioxidant tests are expressed as *µ*mol Trolox equivalent (TE) per gram.

#### 2.4.2. ABTS Method

3 mL of ABTS^+^ solution was mixed with 10 *μ*L of the extracts. A decrease in absorbance was measured at *λ* = 734 nm [12].

#### 2.4.3. CUPRAC Assay

The CUPRAC solution encompassed CuCl_2_ × 2H_2_O (0.01 M in water), ammonium acetate buffer solution (0.001 M, pH = 7), and neocuproine (0.0075 M in ethanol) (1 : 1 : 1 ratio). 3 ml of CUPRAC reagent was mixed with 10 *μ*L of the extracts. An increase in absorbance was recorded at *λ* = 450 nm [13].

#### 2.4.4. FRAP Assay

The FRAP solution encompassed TPTZ (0.01 M dissolved in 0.04 M HCl), FeCl_3_ × 6H_2_O (0.02 M in water), and acetate buffer (0.3 M, pH 3.6) (1 : 1 : 10 ratio). 3 mL of freshly prepared FRAP reagent was mixed with 10 *μ*L of the extracts. An increase in absorbance was recorded at *λ* = 593 nm [14].

### 2.5. Statistical Analysis

All spectrophotometrical experiments were performed in triplicate, and their results were expressed as an average ± standard deviation. Statistically significant differences between the examined propolis extracts were determined by applying the one-way ANOVA method and using SPSS 25.0 software (Chicago, IL, USA).

## 3. Results and Discussion

### 3.1. Identification and Quantification of Phenolic Compounds by UHPLC-ESI-MS/MS

Since the applicability of conventionally produced ethanolic propolis to certain groups of consumers (e.g., children and patients with chronic diseases) is limited, the options of producing nonethanolic extracts, which would be comparable to (as effective as) ethanolic extracts in terms of their chemical composition and biological effect, are being sought. The production of nonethanolic propolis extracts is becoming a major challenge due to the very limited water solubility of propolis. Efforts are being made to find various technologies to improve the extraction of active compounds of propolis in a nonethanolic solvent. The aim of this research was to prepare nonethanolic propolis extract by using PEG as a solvent and to compare the qualitative and quantitative composition and antioxidant (radical-scavenging and reduction properties) activity of AQUA-PEG and EEP. Separation of compounds of prepared extracts from propolis collected in Lithuania was carried out with Acquity H-class UPLC system equipped with triple quadrupole tandem mass spectrometer with an electrospray ionization source (ESI) to obtain MS/MS data. Mass spectrometry parameters for the analysis of phenolic compounds and quinic acid are shown in Table 1.

Table 2 illustrates the chemical composition of flavonoids in AQUA-PEG and EEP. It is important to note that, in the case of 23 phenolic acids and flavonoids identified on the basis of the HPLC method, the total (sum) content thereof was very similar (433.53 *µ*g/mL in AQUA-PEG and 400.36 *µ*g/mL in EEP). By using a nonethanolic solvent, its extraction extent was 1.31-fold greater in the case of phenolic acids than that in ethanolic solvent, whereas the extraction of flavonoids was(2.38-fold greater in the case of ethanol than that in nonethanolic solvent. The UPLC-ESI-MS/MS analysis showed that the largest group of compounds identified in AQUA-PEG and EEP were flavonoids, namely, isorhamnetin, apigenin, quercetin, hyperoside, and kaempferol, and their content varied between 2.02 and 14.03 *µ*g/mL in AQUA-PEG and between 6.04 and 37.5 *µ*g/mL in EEP (Table 2). The content of isorhamnetin, apigenin, quercetin, and kaempferol in EEP was approximately 2–3-fold higher than that in AQUA-PEG, whereas the content of hyperoside was similar (Table 2). The following flavonoids—rutin, galangin, isorhamnetin-3-O-rutinoside, isorhamnetin-3-O-glucoside, kaempferol-3-O-glucoside, catechin, avicularin, phloridzin, and vitexin—were also identified, although their content was much lower and varied between 0.012 and 0.32 *µ*g/mL in AQUA-PEG and between 0.014 and 1.12 *µ*g/mL in EEP (Table 2). The content of galangin and isorhamnetin-3-O-rutinoside was 14 and 16 times higher in EEP than that in AQUA-PEG, respectively, and the content of isorhamnetin-3-O-glucoside, kaempferol-3-O glucoside, avicularin, and phloridzin identified was also 2–5 times higher in EEP than that in AQUA-PEG. Orientin and tiliroside were not identified in AQUA-PEG at all, only in EEP (Table 2). Thus, the total content of the flavonoids identified in EEP was 2.3-fold higher (72.5 *µ*g/mL) than that in AQUA-PEG (30.9 *µ*g/ml).

Comparing the obtained results with the data of others, the peculiarities of the composition of propolis samples collected in Lithuania were determined. Fabris et al. found that galangin is one of the major phenolic compounds in propolis collected in Dagestan [15]. The concentration of galangin was low in propolis samples collected in Lithuania and comparable to the concentration of this compound in propolis samples, collected in Sochi region, Russia [15]. Quercetin is one of the dominant compounds among flavonoids in Lithuanian propolis samples, while the study of Kalogeropoulos et al. found only low concentrations of this compound in propolis collected in Greece and Cyprus [16].

Moreover, 5 phenolic acids (p-coumaric, caffeic, sinapic, chlorogenic, and rosmarinic acid) were determined in both EEP and AQUA-PEG (Table 3). The total content of the identified phenolic acids and their derivatives was 430.44 *µ*g/mL in AQUA-PEG and 326.87 *µ*g/mL in EEP. It is important to note that the content of caffeic, sinapic, and chlorogenic acids was 3–5 times higher in AQUA-PEG than that in EEP (Table 3), the content of rosmarinic acid was similar, and the content of p-coumaric acid was even higher (1.44-fold) in EEP. The most predominant compound of all the analytes identified and quantified was p-coumaric acid, followed by caffeic acid and sinapic acid (Table 3). Moreover, quinic acid was detected in both AQUA-PEG and EEP. The quantity of quinic acid was 1.52-fold higher in AQUA-PEG.

Hegazi et al., studying the chemical composition of propolis collected in Austria, Germany, and France, showed that p-coumaric acid predominated among all phenolic acids [17]. These research data are in agreement with our research findings. Samples of propolis collected in Lithuania were also dominated by p-coumaric acid. Al-Ani et al. provided slightly different research data. In the European-type propolis samples studied by these researchers, p-coumaric acid was qualitatively detected only in the propolis samples collected in the Czech Republic, while it was not identified at all in the propolis samples collected in Germany and Ireland [18].

### 3.2. Measurements of Antioxidant Activity in Extracts

The positive effect of biologically active compounds with antioxidant activity on the body has been proven by the results of numerous scientific research [19, 20]. One of the most important natural compounds with strong antioxidant [21], anti-inflammatory [22], antiallergic [23], antibacterial [24], antiviral [25], antithrombogenic [26], anticancer [27], and cardiovascular system-protective effects [28] is phenolic compounds. An association between the intake of food rich in phenolic compounds and morbidity in the case of oncological [29], cardiovascular [30], and neurodegenerative diseases [31] has been established.

Therefore, the evaluation of the antioxidant activity of the extracts derived from propolis raw materials is of crucial importance. This provides a scientific basis for the use of propolis preparations for the prevention and treatment of diseases caused by oxidative stress. Natural extracts encompass a variety of compounds that act as antioxidants through different reaction mechanisms [28, 29]. It is noted in the scientific literature that the performance of a single test to evaluate antioxidant activity may not be sufficient, because it depends on experimental conditions, methods used, and so on; therefore, it is recommended to apply at least two different methods [32]. In order to obtain the most comprehensive and accurate results possible, it is crucial to identify which methods for the determination of antioxidant activity *in vitro* best reflect the antiradical and reduction effects of biologically active compounds with a different structure and properties that are found in propolis. For this reason, at the time of determining antioxidant activity, the antiradical activity of EEP and AQUA-PEG was evaluated using 4 different *in vitro* methods (ABTS, CUPRAC, DPPH, and FRAP). The ABTS and DPPH assays are based on the antioxidants' ability to scavenge free radicals [11, 12], whereas the FRAP and CUPRAC assays measure the reduction activity of antioxidants [13, 14].

Despite a significant difference in the content of biologically active compounds (phenolic acids and flavonoids) in EEP, as compared to AQUA-PEG, the antiradical activity (ABTS method) and reduction properties (CUPRAC method) are quite similar in the case of both extracts. However, the evaluation of the reduction activity of the examined propolis extracts *in vitro*, through the application of the spectrophotometric FRAP method, revealed that the reduced activity in the ethanolic extract was 2 times stronger than that in the AQUA-PEG extract (Table 4, *p* < 0.05). What is more important, the evaluation of the antiradical activity of these extracts by applying the DPPH method revealed that the EEP was characterized by as much as 12 times stronger DPPH-free radical-scavenging activity than the AQUA-PEG (Table 4, *p* < 0.05). Thus, it clearly shows that antioxidant activity is highly dependent on the methods used to assess antioxidant activity and can vary greatly depending on the method. This should be taken into account at the time of research. Nevertheless, the following question remains: what are the reasons for such differences in the antioxidant activity of propolis extracts.

Thus, the results of this study show that the antioxidant activity depends on the method used: in the case of the ABTS method, it is 5-fold higher in AQUA-PEG and 3-fold higher in EEP than that in the case of the FRAP method. It would appear that, due to the methodological conditions (acidic medium), the antioxidant activity of some propolis compounds decreases. This should be taken into account when conducting research and applying different methods, as well as interpreting the results. The research conducted by the authors of this study is significant in that it demonstrates the advantages and disadvantages of various antioxidant-related methods. Thus, the authors deem it appropriate to apply several different methods in performing the assessment of multicomponent extracts, especially such as propolis, and especially in those cases when the extracts encompass not only hydrophobic but also hydrophilic compounds. To the knowledge of the authors, this is the first study that compares the antioxidant activity of propolis on the basis of as many as four different methods.

It should be noted that DPPH-free radicals are soluble only in organic solvents, which limits the evaluation of the antiradical activity of hydrophilic antioxidants on the basis of this method. When ethanol is used for extraction, more phenolic compounds with lipophilic properties are extracted, which explains why the antiradical activity of EEP *in vitro* determined on the basis of the DPPH method is significantly stronger than that in the case of AQUA-PEG. The results of this study showed that the quantity of hydrophilic compounds (caffeic, sinapic, and chlorogenic acids) was as much as 3–5 times higher in AQUA-PEG than that in EEP (Table 1), whereas the quantity of various flavonoids was much higher (2.3-fold higher) in EEP than that in AQUA-PEG (Table 2). The quantity of isorhamnetin, apigenin, quercetin, and kaempferol in EEP was about 2-3 times higher and the quantity of galangin and isorhamnetin-3-O-rutinoside was 14 and 16 times higher than those in AQUA-PEG.

It is also important to note that the association between the structure of phenolic compounds and their antioxidant activity in biological systems or living organisms is much more complex because the antioxidant activity of each compound is highly dependent on its physicochemical properties, such as lipophilicity, solubility, and distribution between lipophilic and hydrophilic medium. Propolis is a natural multicomponent product, in the extracts of which phenolic compounds with different lipophilicity are characterized by the antioxidant effect of unequal strength and various mechanisms of action.

## 4. Conclusion

In summary, the results of the present study show that the total quantity of phenolic compounds in both EEP and AQUA-PEG is similar but the antioxidant activity differs significantly, which depends on the method used for the antioxidant-related research. By applying the ABTS and CUPRAC methods, both extracts—EEP and AQUA-PEG—demonstrate similar antioxidant (antiradical and reduction) activity, whereas in the case of the DPPH and FRAP method, significantly higher antioxidant activity was detected in EEP. This should be taken into account especially when interpreting the results of the studies on the properties of propolis and drawing conclusions about the antioxidant activity of propolis extracts. Nevertheless, the important part of the findings made by the authors of this study is that polyethylene glycol is quite a good enough solvent, which can be successfully used for the preparation of nonethanolic propolis extracts, especially in cases where ethanolic propolis extracts should not be used (for children and also for people who are sensitive to ethanol, etc.). On the basis of the results of the present study, both extracts of propolis—ethanolic and nonethanolic—can be used as a functional food or as a dietary supplement encompassing biologically active compounds, since both ethanolic and nonethanolic propolis extracts contain a large quantity of antioxidants, namely, flavonoids and phenolic acids. The authors of this study believe that this has the prospect of being applied as an additional measure both in the prevention of diseases and in the alleviation of certain symptoms under conditions of oxidative stress.

## Figures and Tables

**Table 1:** Mass spectrometry parameters for the analysis of phenolic compounds and quinic acid.

Compound	Parent ion (*m*/*z*)	Daughter ion (*m*/*z*)	Cone voltage (V)	Collision chamber energy (*e*V)
p-Coumaric acid	163	93	28	22
Caffeic acid	179	107	36	22
Quinic acid	191	85	40	26
Sinapic acid	223	208	32	14
Apigenin	269	117	54	36
Galangin	269	171	50	30
Kaempferol	285	185	50	25
(+)-Catechin	289	123	60	34
Quercetin	301	151	48	20
Isorhamnetin	315	300	44	22
Chlorogenic acid	353	191	32	14
Vitexin	431,1	311	50	22
Avicularin	433	301	50	20
Phloridzin	435	273	42	14
Kaempferol-3-O-glucoside	447	284	54	28
Orientin	447	327	50	20
Hyperoside	463	300	50	26
Isorhamnetin-3-O-glucoside	477	314	60	28
Tiliroside	593,1	285	64	30
Rutin	609	300	70	38
Isorhamnetin-3-O-rutinoside	623	315	70	32
Rosmarinic acid	359	161	36	16

**Table 2:** Content (*µ*g/ml) of flavonoids in AQUA-PEG and EEP.

	AQUA-PEG (*µ*g/mL)	EEP (*µ*g/mL)
Isorhamnetin	14.0 ± 0.6	37.5 ± 1.8
Apigenin	6.5 ± 0.3	13.7 ± 0.6
Quercetin	3.8 ± 0.2	8.6 ± 0.4
Hyperoside	3.3 ± 0.1	3.7 ± 0.2
Kaempferol	2.0 ± 0.1	6.0 ± 0.2
Rutin	0.32 ± 0.02	0.38 ± 0.02
Galangin	0.08 ± 0.004	1.12 ± 0.05
Isorhamnetin-3-O-rutinoside	0.07 ± 0.003	1.12 ± 0.06
Kaempherol-3-O-glucoside	0.17 ± 0.01	0.46 ± 0.02
Isorhamnetin-3-O-glucoside	0.13 ± 0.01	0.28 ± 0.01
(+)-Catechin	0.22 ± 0.01	0.06 ± 0.003
Avicularin	0.10 ± 0.01	0.31 ± 0.02
Phloridzin	0.03 ± 0.002	0.15 ± 0.01
Vitexin	0.012 ± 0.001	0.014 ± 0.001
Orientin	ND	0.036 ± 0.002
Tiliroside	ND	0.037 ± 0.002

**Table 3:** Content (*µ*g/mL) of phenolic acids in AQUA-PEG and EEP.

	AQUA-PEG (*µ*g/mL)	EEP (*µ*g/mL)
p-Coumaric acid	164.0 ± 7.8	236.7 ± 10.2
Caffeic acid	136.7 ± 6.0	45.8 ± 2.0
Sinapic acid	129.5 ± 5.71	43.1 ± 1.9
Chlorogenic acid	40.0 ± 1.9	7.6 ± 0.3
Rosmarinic acid	0.24 ± 0.01	0.33 ± 0.02

**Table 4:** Antioxidant activity *in vitro* of propolis extracts.

Method	Antioxidant activity *in vitro µ*mol (TE/g)	
	AQUA-PEG	EEP
ABTS	66.0 ± 1.8	64.6 ± 3.0
CUPRAC	56.9 ± 3.4	57.8 ± 1.2
DPPH	107.1 ± 10.1	1299.5 ± 43.9
FRAP	10.7 ± 1.2	21.3 ± 4.2

## Data Availability

The data used to support the findings of this study are included within the article.

## Abbreviations

ABTS: 2,2′-Azino-bis(3-ethylbenzthiazoline-6-sulfonic acid)
CUPRAC: Cupric reducing antioxidant capacity
DPPH: 2,2-Diphenyl-1-picrylhydrazyl
EEP: Ethanolic propolis extract
ESI: Electrospray ionization source
FRAP: Ferric reducing antioxidant power
LC-MS: Liquid chromatography-mass spectrometry
MS/MS: Tandem mass spectrometry
*m*/*z*: Mass-to-charge ratio
NEP: Nonethanolic propolis extract
PEG: Polyethylene glycol
TE: Trolox equivalent
TPTZ: 2,4,6-Tripyridyl-s-triazine
UHPLC: Ultrahigh-performance liquid chromatography
UHPLC-ESI-MS/MS: Ultrahigh-performance liquid chromatography-electrospray tandem mass spectrometry
UV/VIS: Ultraviolet-visible.
